# Pathway-PDT: a flexible pathway analysis tool for nuclear families

**DOI:** 10.1186/1471-2105-14-267

**Published:** 2013-09-04

**Authors:** Yo Son Park, Michael Schmidt, Eden R Martin, Margaret A Pericak-Vance, Ren-Hua Chung

**Affiliations:** 1 John P. Hussman Institute for Human Genomics, University of Miami Miller School of Medicine, Miami, FL, USA; 2 Dr. John T. Macdonald Foundation Department of Human Genetics, University of Miami Miller School of Medicine, Miami, FLUSA; 3 Division of Biostatistics and Bioinformatics, Institute of Population Health Sciences, National Health Research Institutes, Zhunan, Miaoli, Taiwan

**Keywords:** Pathway, GWAS, PDT, GSEA, Kolmogorov-Smirnov-like running sum statistic, Pedigree GWAS

## Abstract

**Background:**

Pathway analysis based on Genome-Wide Association Studies (GWAS) data has become popular as a secondary analysis strategy. Although many pathway analysis tools have been developed for case–control studies, there is no tool that can use all information from raw genotypes in general nuclear families. We developed Pathway-PDT, which uses the framework of Pedigree Disequilibrium Test (PDT) for general family data, to perform pathway analysis based on raw genotypes in family-based GWAS.

**Results:**

Simulation results showed that Pathway-PDT is more powerful than the p-value based method, ALIGATOR. Pathway-PDT also can be more powerful than the PLINK set-based test when analyzing general nuclear families with multiple siblings or missing parents. Additionally, Pathway-PDT has a flexible and convenient user interface, which allows users to modify their analysis parameters as well as to apply various types of gene and pathway definitions.

**Conclusions:**

The Pathway-PDT method is implemented in C++ with POSIX threads and is computationally feasible for pathway analysis with large scale family GWAS datasets. The Windows binary along with Makefile and source codes for the Linux are available at https://sourceforge.net/projects/pathway-pdt/.

## Background

Genome-wide association studies (GWAS) have been successful in identifying single nucleotide polymorphisms (SNPs) associated with complex diseases [1,2]. Almost a million or even several millions of SNPs are densely genotyped across the genome for GWAS, and single-SNP association tests are performed to identify individual SNPs with marginal effects on the disease. Nonetheless, GWAS may lose power for identifying disease loci due to the stringent significance threshold required for multiple testing correction for the multitude of SNPs tested [3]. For a complex disease that is expected to be caused by the joint effects of multiple genes, statistical power can be increased if the joint effects are considered in the test.

Statistical pathway analysis based on GWAS data has become a popular secondary analysis strategy [4-6]. Combining single-SNP association tests within a pathway in statistical tests can help identify the joint effects of genetic variations underlying complex disease susceptibility that were difficult to find using the single-SNP association tests alone. Current pathway analysis approaches can be classified into two types, the self-contained test and the competitive test, based on their null hypotheses [7]. The self-contained test compares the test statistics for genes in a given pathway to the test statistics for the same genes under the null. The competitive test compares the test statistics for genes in a given pathway to test statistics for other genes in the genome [8]. Either SNP p-values or raw genotypes are expected by current pathway analysis methods for GWAS. The advantages for p-value based methods (i.e. methods accepting SNP p-values) include the flexibility for accommodating different study designs such as unrelated case–control and family-based studies. Moreover, data sharing for a list of p-values is easier than individual raw genotypes when a joint analysis is performed to combine different datasets in a consortium. The major advantage for raw-genotype based methods is that permutations can be performed (by either permuting phenotypes or genotypes under the null) to account for linkage disequilibrium (LD) structures among SNPs and for different gene and pathway sizes [8].

Most of the currently available software packages for pathway analysis are designed for case–control studies or are restricted to use trios (two parents and one affected sib) [4,9,10]. PLINK [11] provides a set-based test based on the Transmission Disequilibrium Test (TDT) [12] statistics, which can be used for family-based pathway association analysis. The set-based test is restricted to use trio families with parents, as parental genotypes are required for the TDT statistics calculations. The user has to use external bioinformatics tools such as the UCSC genome browser [13] to map SNPs to genes and generate a set of SNP IDs in a pathway for the set-based test, which can increase analysis difficulty. GenGen, implementing Wang’s method [10], is another tool for family-based pathway analysis. GenGen, which is also based on the TDT statistics, has the same restriction of using trio families. Several complex disease studies involve nuclear families with multiple affected and unaffected siblings, such as the autism GWAS data publicly available through the Autism Genetic Resources Exchange (AGRE) consortium (http://research.agre.org). Moreover, for late-onset diseases such as Alzheimer disease, parental genotypes are often missing. Statistical power for the PLINK set-based test and the GenGen test can be reduced for analyzing such families.

Another way to perform pathway analysis for general nuclear families, such as families with multiple siblings or missing parents, is to use the p-value based methods. The first step for the analysis is to obtain p-values from existing family-based single-SNP association tests that can accommodate general nuclear family structures such as the Pedigree Disequilibrium Test (PDT) [14] and FBAT [15]. Then the set of p-values is provided to a p-value based method as the second step. However, statistical power could be compromised when a p-value based method is used rather than a raw-genotype based method [16]. Moreover, it would be ideal to integrate all of the pathway analysis steps into a single efficient computer program.

Here, we integrated two well-established algorithms, the PDT and the modified gene set enrichment analysis (GSEA) [17] algorithm as proposed in Wang et al. [10], into a family-based pathway analysis method. The software implementing the method, Pathway-PDT, can use nuclear families with one or more affected siblings and allows for missing parents. The Pathway-PDT software is implemented in C++ and uses threads for parallel processing of multiple permutations to increase the computational efficiency.

## Implementation

### The Pathway-PDT algorithm

The Pathway-PDT algorithm combines the framework of the family-based association test, PDT [14], and the weighted Kolmogorov-Smirnov-like (KS-like) running sum statistic proposed in GSEA for gene expression analysis [17] and its GWAS adaptation first suggested in the Wang et al. study [18]. Pathway-PDT inherits the properties of PDT that it can use general nuclear families with multiple affected and unaffected siblings and allow for missing parents. The KS-like test compares the distributions of gene scores for genes within and outside a given pathway. Therefore, Pathway-PDT is a competitive test that uses genome-wide information for testing a pathway.

There are several steps in the Pathway-PDT algorithm:

(1) Assign SNPs to genes. SNPs are assigned to a gene if they are inside the gene or *k* kb away from the gene. The parameter *k* is specified by the user. A commonly used *k* is 5 kb or 20 kb to account for SNPs in regulatory regions for the gene.

(2) Calculate PDT statistic for each SNP that has been assigned to a gene.

(3) For each gene, select the largest PDT statistic (corresponding to the minimum p-value) from the PDT statistics for all SNPs assigned to the gene as a score for the gene.

(4) Let the total number of genes in the dataset be *N*, where the *j*th gene, *G*_*j*_, has a score *r*_*j*_. The *N* genes are sorted by their gene scores from largest to smallest. For each pathway *P*, calculate the weighted KS-like running sum statistic (referred to as the Enrichment Score of *P* or *ES(P)*) by the following [17]:

$$\mathrm{ES}\left(P\right)=\underset{1\leq i\leq N}{\text{max}}\left\{\underset{G_{j}\in P,j\leq i}{\sum }\frac{{\left|r_{j}\right|}^{w}}{N_{R}}-\underset{G_{j}\notin P,j\leq i}{\sum }\frac{1}{N-N_{H}}\right\}$$

where $N_{R}=\sum_{G_{j}\in P}{\left|r_{j}\right|}^{w}$ , *w* is the weight for each gene and *N*_*H*_ is the number of genes in *P*. The default weight *w* is 1 for Pathway-PDT as recommended in the GSEA algorithm [17].

(5) Permute the transmitted and untransmitted alleles from parents to siblings within each family and recalculate the PDT statistics for SNPs within genes.

(6) Repeat steps 3–5 for *K* times.

The p-value for the Pathway-PDT test is the proportion of times that the permuted *ES(P)* is greater than the observed *ES(P)* in the *K* times. Based on our simulation results, Pathway-PDT maintained correct type I error rates when *K* was specified as 2,000. However, a larger number of *K* is required if a higher precision of p-value is needed. The null hypothesis is that the distribution of gene scores in *P* is the same as the distribution of gene scores for other genes in the genome.

Similar to Monks and Kaplan [19], it can be shown that permuting the transmitted and untransmitted alleles from parents to siblings within a family results in a sign change for the PDT statistic for the family. Therefore, even when parents are missing in a family, permuting the PDT statistic is still possible by simply changing the sign of the statistic for the family. Alleles at SNPs on the same chromosome are permuted simultaneously to preserve the LD structures among the SNPs. Note that calculating the PDT statistics in a permutation requires raw genotypes or the PDT statistic for each of the families. The information cannot be obtained from single-SNP p-values or single-SNP statistics. Also the statistics are recalculated based on the same sizes of genes and pathways as the original sizes in each permutation. Therefore, the permutation procedure properly accounts for gene and pathway sizes so that large genes or pathways do not bias the Pathway-PDT statistic under the null. Pathway-PDT maintains the advantage of raw-genotype based method that LD structures, gene sizes, and pathway sizes are properly accounted for in the test. Moreover, the permutation statistics are used to calculate the permutation-adjusted p-values and False Discovery Rate (FDR) [20] in Pathway-PDT to adjust results for multiple testing corrections.

### Comparison between Wang’s method and Pathway-PDT

Both Wang’s method [10] and Pathway-PDT were extended from the GSEA algorithm. As discussed in Wang et al., their method can be applied to unrelated case–control or family-based studies. The GenGen package provided by the authors uses the TDT statistics as the fundamental single-SNP statistics. Therefore, the software is restricted to analysis of trios. The procedure of calculating the TDT statistics and the permuted statistics (i.e. calculate_association.pl), and the procedure of calculating the pathway statistics (i.e. calculate_gsea.pl) are implemented in two different Perl scripts in GenGen. In order to improve the permutation efficiency for a large number of permutations (e.g. 2,000 permutations), the user has to split the permutations into several parts (e.g. 10 parts, each part has 200 permutations), run calculate_association.pl to calculate the permuted statistics for each of the parts in parallel, and provide all the files containing the permuted statistics to calculate_gsea.pl to obtain the final pathway results, as suggested in the user manual. In contrast, the Pathway-PDT method is developed based on the PDT statistic, which can use general nuclear families with missing parents and multiple affected siblings. Even when parents are missing in a family, calculating the permuted PDT statistic is still possible by simply permuting the sign of the original PDT statistic. The procedures of calculating the PDT and Pathway-PDT statistics, and the permutation procedures are automatically performed in Pathway-PDT in a single run. Moreover, threads can be used to parallelize the permutation procedures on a computer with multi-core CPUs. Therefore, Pathway-PDT provides an efficient and user-friendly tool for family-based GWAS pathway analysis.

### Simulations for power and type I error calculations

Simulation studies were conducted to evaluate the type I error rates and to compare power for the Pathway-PDT with the p-value based tool, ALIGATOR, which uses p-values from GWAS and a bootstrap sampling approach to estimate empirical p-values, and the PLINK set-based test, which is a raw-genotype based test. The PLINK set-based test has been shown to be a powerful test for pathway analysis using simulations and real data applications [9,16]. The single-SNP PDT p-values were used as input for ALIGATOR.

A forward-time population simulation program, genomeSIMLA [21] was used to simulate GWAS datasets based on the Illumina HumanHap550 genotyping chip and the LD information of the HapMap CEU population. No causal variants were simulated for the type I error analysis. Disease models for the power analyses included two additive models (Model1 and Model2) with ten causal variants. The minor allele frequencies for the ten variants were close to 0.2. The odds ratios for the ten variants were 1.2 and 1.3 for Model 1 and Model 2, respectively, following the estimated average effect size of common variants contributing to the complex disease susceptibility [22]. A pathway with 24 genes, which contain 285 variants, was used to evaluate both type I error rates and power. A total of 2,000 and 500 replicates were generated to calculate type I error rates and power, respectively. A total of 500 nuclear families (two parents and three siblings, where at least one sibling is affected) were simulated for each replicate. For scenario 1, we assumed all 500 families had parents. For scenario 2, we assumed 50% of the families had missing parents. Gene weights were set to be 1 as used by GSEA and Wang et al. study [10,17]. Default parameters in PLINK and ALIGATOR were used for the simulations.

## Results and discussion

**Figure 1:**
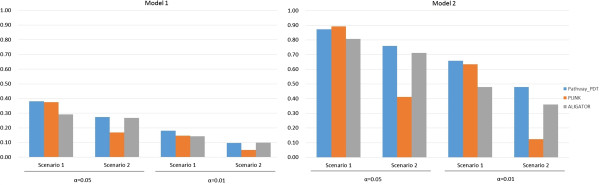
**Power comparisons of Pathway-PDT with the PLINK set-based test and ALIGATOR.** Disease models for the power analyses included two additive models, Model 1 and Model 2, with 10 causal variants at minor allele frequencies near 0.2 and odds ratios of 1.2 and 1.3, respectively. A pathway with 24 genes, which contain 285 variants, for a total of 500 replicates of 500 nuclear families (two parents and three siblings, where at least one sibling is affected) was used to evaluate power. For scenario 1, we assumed all 500 families had parents. For scenario 2, we assumed 50% of the families had missing parents. Gene weights were set to be 1 as used by GSEA and Wang et al. study [17,18]. Default parameters in PLINK and ALIGATOR were used for the simulations.

The null data simulations showed that type I error rates were controlled for Pathway-PDT at the 0.05 and 0.01 significance levels. Figure 1 shows the power comparison of Pathway-PDT with PLINK and ALIGATOR at the 0.05 and 0.01 significance levels. The power patterns are similar for Models 1 and 2. Pathway-PDT is generally the most powerful test, except for Model 2 under scenario 1 where all families have parents, PLINK is the most powerful test at the 0.05 significance level. While not comprehensive, the simulation results suggest that Pathway-PDT, which directly uses raw genotypes in families, can be more powerful than the p-value based method, ALIGATOR. When families have more than one sibling or missing parents, Pathway-PDT can also have more power than the PLINK set-based test. This again demonstrates the importance of the implementation of the Pathway-PDT method for family-based pathway analysis.

Pathway-PDT requires three types of input files: a gene file, which contains the locations of genes, a pathway file with pathway definitions, and the standard PLINK map and ped files. The analysis of pathways is performed in parallel using the POSIX threads (pthreads). The total run time of Pathway-PDT for analyzing 210 KEGG pathways for 1,000 permutations based on a GWAS dataset with 710 families genotyped on the Illumina 1 M chip platform was 42 minutes on 8 Intel ×86-64 processors. Therefore, Pathway-PDT can efficiently perform large-scale pathway analysis in a reasonable time frame.

## Conclusion

In conclusion, we implemented a family-based pathway algorithm, Pathway-PDT, in an efficient software package. The routine procedures of pathway analysis such as mapping SNPs to genes and mapping genes to pathways, the procedures of calculating the single-SNP and pathway statistics, and calculating the permutation adjusted p-values and FDR are automatically performed in the Pathway-PDT software in a single run. Moreover, threads are used to run the permutations in parallel to increase the efficiency of the tool. With convenient user interface and efficient performance, Pathway-PDT will be very useful for analyzing family-based GWAS datasets.

## Availability and requirements

**Project name:** Pathway-PDT

**Project home page:**https://sourceforge.net/projects/pathway-pdt/

**Operating system(s):** Windows and Linux

**Programming language:** C++ with POSIX threads (pthreads)

**Restrictions on use by non-academics:** no limitations

## Competing interests

The authors declare that they have no competing interests.

## Authors’ contributions

YSP was the primary author on the manuscript. YSP, MS, and RHC developed the Pathway-PDT method and software, and tested the program intensively on simulated datasets. ERM and MPV provided input to study design. All authors contributed to writing of the manuscript. All authors read and approved the final manuscript.
